# The role of *RB1* alteration and 4q12 amplification in IDH-WT glioblastoma

**DOI:** 10.1093/noajnl/vdab050

**Published:** 2021-03-31

**Authors:** Antonio Dono, Arvind V Ramesh, Emily Wang, Mauli Shah, Nitin Tandon, Leomar Y Ballester, Yoshua Esquenazi

**Affiliations:** 1 Vivian L. Smith Department of Neurosurgery, McGovern Medical School, The University of Texas Health Science Center at Houston, Houston, Texas, USA; 2 Department of Pathology and Laboratory Medicine, McGovern Medical School, The University of Texas Health Science Center at Houston, Houston, Texas, USA; 3 Center for Precision Health, School of Biomedical Informatics, The University of Texas Health Science Center at Houston, Houston, Texas, USA; 4 Rice University, Houston, Texas, USA; 5 Memorial Hermann Hospital-TMC, Houston, Texas, USA

**Keywords:** glioblastoma, IDH-WT, *KDR*, *RB1*, *VEGFR2*, 4q12

## Abstract

**Background:**

Recent studies have identified that glioblastoma IDH-wildtype (GBM IDH-WT) might be comprised of molecular subgroups with distinct prognoses. Therefore, we investigated the correlation between genetic alterations and survival in 282 GBM IDH-WT patients, to identify subgroups with distinct outcomes.

**Methods:**

We reviewed characteristics of GBM IDH-WT (2009–2019) patients analyzed by next-generation sequencing interrogating 205 genes and 26 rearrangements. Progression-free survival (PFS) and overall survival (OS) were evaluated with the log-rank test and Cox regression models. We validated our results utilizing data from cBioPortal (MSK-IMPACT dataset).

**Results:**

Multivariable analysis of GBM IDH-WT revealed that treatment with chemoradiation and *RB1-*mutant status correlated with improved PFS (hazard ratio [HR] 0.25, *P* < .001 and HR 0.47, *P* = .002) and OS (HR 0.24, *P* < .001 and HR 0.49, *P* = .016). In addition, younger age (<55 years) was associated with improved OS. Karnofsky performance status less than 80 (HR 1.44, *P* = .024) and *KDR* amplification (HR 2.51, *P* = .008) were predictors of worse OS. *KDR-*amplified patients harbored coexisting *PDGFRA* and *KIT* amplification (*P* < .001) and *TP53* mutations (*P* = .04). *RB1-*mutant patients had less frequent *CDKN2A/B* and *EGFR* alterations (*P* < .001). Conversely, *RB1-*mutant patients had more frequent *TP53* (*P* < .001) and *SETD2* (*P* = .006) mutations. Analysis of the MSK-IMPACT dataset (*n* = 551) validated the association between *RB1* mutations and improved PFS (11.0 vs 8.7 months, *P =* .009) and OS (34.7 vs 21.7 months, *P =* .016).

**Conclusions:**

*RB1*-mutant GBM IDH-WT is a molecular subgroup with improved PFS and OS. Meanwhile, 4q12 amplification (*KDR/PDGFRA/KIT)* denoted patients with worse OS. Identifying subgroups of GBM IDH-WT with distinct survival is important for optimal clinical trial design, incorporation of targeted therapies, and personalized neuro-oncological care.

**Key Points**

- * RB1-*mutant GBM IDH-WT patients have improved PFS and OS.
- * RB1-*mutant GBM IDH-WT patients have a lower frequency of *CDKN2A/B* loss and *EGFR* alterations.
- 4q12-amplified GBM IDH-WT patients have worse survival.

**Importance of the Study**

Glioblastoma IDH-wildtype (GBM IDH-WT) comprises different molecular subgroups; however, the prognostic significance of these subgroups has not been defined. We demonstrated in a large molecularly characterized GBM IDH-WT cohort 2 genetically distinct subgroups with different prognoses. Our findings were validated with large external GBM IDH-WT datasets. GBM IDH-WT with *RB1* mutations is a molecular subgroup with improved PFS and OS and decreased frequency of *CDKN2A/B* loss and *EGFR* alterations. On the other hand, 4q12 (*KDR/PDGFRA/KIT*) amplified patients have worse survival. Our data revealed the importance of genetic profiling of GBM IDH-WT to identify subgroups with distinct survival. This is crucial for optimal clinical trial design, targeted therapies, and personalized neuro-oncological care.

Glioblastoma (GBM) is the most common and aggressive central nervous system (CNS) primary malignancy.^1^ Despite aggressive treatment with maximal safe resection and chemoradiotherapy,^2^ and multimodal therapy upon recurrence, GBM is associated with a dismal prognosis.^1^

Over the past decade, molecular characterization of gliomas has revealed the heterogeneous nature of this group of tumors.^3–7^ These findings led to a reclassification of infiltrating gliomas, in which both tumor histology and genetic alterations are considered.^8^ Infiltrating gliomas are classified by the presence or absence of mutations in the isocitrate dehydrogenase (IDH) 1 or 2 genes, as IDH-wildtype (WT) or IDH-mutant, with different demographic, clinical, and prognostic characteristics.^8^ Recent studies have identified that GBM IDH-WT consists of different molecular subgroups, which might have a distinct prognosis.^6,7,9,10^ However, more studies are needed to understand molecular subgroups of GBM IDH-WT as survival differences between these have not been thoroughly investigated.

Therefore, we examined the correlation between genetic alterations and survival in a cohort of GBM IDH-WT patients, to identify potential subgroups with different behavior, who may potentially benefit from targeted therapies. Our findings were validated using a large publicly available dataset from Memorial Sloan Kettering Cancer Center (MSK-IMPACT).^11^

## Methods

### Patients and Tumor Samples

We performed a retrospective review of GBMs in an institutional glioma registry of patients diagnosed between 2009 and 2019. The inclusion criteria for this study were confirmed diagnosis of GBM IDH-WT according to the cIMPACT-NOW Update 3^12^ and availability of sequencing data from a comprehensive next-generation sequencing (NGS) assay. A flow diagram selection of the study population is depicted in Supplementary Figure S1.

Data for this study were collected from Memorial Hermann Hospital’s electronic medical records. Data were managed with REDCap electronic data capture tools hosted at the University of Texas Health Science Center at Houston (UTHealth).^13^ These included age, sex, race, Karnofsky performance status (KPS), diagnosis, radiographic extent of resection, treatment strategy, and survival. Tumors were classified by a board-certified neuropathologist following the 2016 WHO Classification of Tumors of the CNS^8^ and cIMPACT-NOW updates. Radiographic extent of resection was classified as gross total resection (GTR), near-total resection (NTR), or subtotal resection as previously described.^14^ Recurrence and therapeutic strategy were determined by individual revision of cases by a multidisciplinary tumor board as previously described.^15^

### Ethical Statement

This study was approved by the Institutional Review Board (ID: HSC-MS-17-0917) of UTHealth and Memorial Hermann Hospital, Houston, TX.

### Targeted Sequencing

Tumor samples were analyzed for genomic alterations by a targeted NGS panel interrogating 205 genes and 26 gene rearrangements including telomerase reverse transcriptase promoter *(TERTp*) mutations (FoundationOne; Foundation Medicine, Inc.). The FoundationOne assay was performed in a Clinical Laboratory Improvement Amendments certified laboratory, as previously described.^16,17^*TERTp* status was not available for 61 patients.

### Validation Cohorts

To validate our findings, we utilized the dataset from the MSK-IMPACT available at cBioPortal (https://www.cbioportal.org/), accessed on August 2020.^11,18,19^ This dataset provided clinical and genetic information including IDH, MGMT, and *TERTp* status. Additionally, we used a GBM cohort with *IDH1* p.R132H status from a publicly available study evaluating the 4q12 amplicon in GBM.^20^ Tumors classified as GBM IDH-WT according to the cIMPACT-NOW Update 3 criteria were analyzed.^12^

### Statistical Analyses

Descriptive analyses were performed by the Mann–Whitney *U* test or Fisher’s exact test for continuous or categorical variables, respectively. The endpoints of the study were overall survival (OS) and progression-free survival (PFS). OS was calculated as the time in months from diagnosis to death or the last available follow-up. PFS was calculated as the time in months from diagnosis to progression of the disease. The univariable two-sided log-rank test was used to examine statistical significance in survival, while the Kaplan–Meier method was employed to plot visual survival curves. Univariable and multivariable Cox proportional hazard regression models were utilized to calculate the hazard ratio (HR) estimates with a 95% confidence interval (CI) adjusted for possible confounders. Multivariable Cox proportional hazard regression model analysis for PFS and OS was adjusted for the variables with a *P* value of .05 or less in univariable analysis, as these might affect survival. Demographic, clinical, and genetic characteristics were evaluated by the genes of interest to identify differences in such traits. The genes of interest were defined as the genes that correlate with survival. *P* value of .05 or less was considered statistically significant and was two-sided. The differences in genetic characteristics were adjusted for multiple comparisons utilizing the Benjamini–Hochberg FDR correction procedure (*q* value). Statistical analyses were performed using EZR (1.40)^17,21^ and Prism v.8.4.3 (GraphPad). The oncoplots were created using cBioPortal OncoPrinter Tool.^18,19^Figures 4 and 5 were created with BioRender.com.

## Results

### Cohort Characteristics

A total of 282 patients with GBM IDH-WT met the inclusion criteria (Supplementary Figure S1). The median age of this cohort was 61 years (interquartile range 53–67.8). One hundred seventy patients (60%) were males, 201 patients (71%) were non-Hispanic White, and 107 patients (38%) had KPS of at least 80. Twenty-seven patients (10%) had a biopsy, while 92 patients (33%) received a GTR. Chemoradiotherapy with temozolomide (TMZ) was administered in 258 (91%) patients according to the Stupp protocol.^2^ Also, 33 (12%) patients were treated with up-front tumor-treating fields (TTFs). Furthermore, 203 patients had documented recurrence of their disease, and out of these patients, 88 (43%) had reoperation for their first recurrence, 94 (46%) received TMZ, 143 (70%) received bevacizumab, 78 (38%) received irinotecan, 17 (8%) lomustine, 63 (31%) TTF, 50 (25%) re-irradiation, and 78 (38%) salvage radiosurgery (Supplementary Table S1).

**Figure 1.**
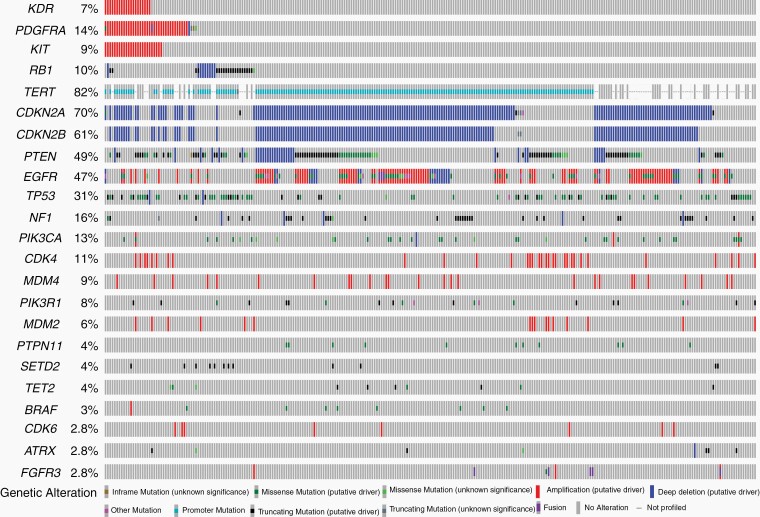
Oncoplot representing the genomic landscape of GBM IDH-WT from UTHealth (*n* = 282).

Genes mutated in at least 3% of patients are depicted in Figure 1 and Supplementary Table S1. The most common genomic alterations in the cohort were in the following genes: *TERTp*—81%, *CDKN2A/B*—70%, *PTEN*—48%, *EGFR*—46%, *TP53*—30%, *NF1*—16%, *PDGFRA*—14%, *PIK3CA*—13%, *CDK4*—11%, *RB1*—10%, *MDM4*—9%, *KIT*—9%, and *KDR*—7%.

### Outcomes in GBM IDH-WT

Univariable analysis of PFS showed that patients who received chemoradiotherapy with TMZ according to the Stupp protocol (HR 0.25, *P* < .001) had a significantly lower risk of death. This finding was further confirmed by multivariable analysis (HR 0.25, *P* < .001; Table 1). Additionally, it was observed that GBM IDH-WT patients harboring *RB1* mutations (*n* = 28) had an improved PFS compared to *RB1-*WT (*n* = 254) patients (11.9 vs 7.5 months, *P =* .0001, log-rank test; Supplementary Figure S2A). This finding was confirmed by multivariable analysis (HR 0.47, *P* = .002; Table 1). The PFS of GBM IDH-WT patients harboring a *KDR* amplification was not significantly different compared to *KDR*-WT patients (5.8 vs 8.3 months, Supplementary Figure S2B).

**Table 1.** Univariable and Multivariable Cox Proportional Hazard Regression Models of Progression-Free Survival and Overall Survival From GBM IDH-WT Patients in the UTHealth Cohort (*n* = 282)

Variable	Univariable	*P*	Multivariable	*P*
	HR (95% CI)		HR (95% CI)	
*Progression-free survival*				
Age at diagnosis <55 years	1.11 (0.83–1.50)	.479		
Male	0.82 (0.62–1.09)	.177		
KPS <80 at diagnosis	0.83 (0.63–1.10)	.191		
Extent of resection				
GTR	1.07 (0.60–1.90)	.822		
NTR	0.96 (0.51–1.80)	.888		
STR	1.09 (0.62–1.91)	.778		
Chemoradiotherapy with TMZ	0.25 (0.13–0.45)	**<.001**	0.25 (0.14–0.48)	**<.001**
*KDR* mutant	1.03 (0.57–1.84)	.935		
*KIT* mutant	1.13 (0.69–1.86)	.625		
*PDGFRA* mutant	1.17 (0.79–1.72)	.438		
*RB1*	0.46 (0.29–0.75)	**.002**	0.47 (0.29–0.76)	**.002**
*TP53* mutant	1.00 (0.74–1.36)	.993		
*Overall survival*				
Age at diagnosis <55 years	0.59 (0.43–0.80)	**<.001**	0.63 (0.45–0.90)	**.010**
Male	0.94 (0.72–1.23)	.670		
KPS <80 at diagnosis	1.63 (1.24–2.15)	**<.001**	1.44 (1.04–1.98)	**.024**
Extent of resection				
GTR	0.59 (0.37–0.94)	**.028**	1.17 (0.62–2.22)	.626
NTR	0.52 (0.31–0.88)	**.015**	1.01 (0.50–2.03)	.975
STR	0.64 (0.41–1.01)	.056	1.13 (0.60–2.13)	.697
Biopsy	Ref.		Ref.	
Chemoradiotherapy with TMZ	0.32 ((0.19–0.53)	**<.001**	0.24 (0.12–0.50)	**<.001**
Second surgery	0.74 (0.54–1.02)	.062		
Salvage bevacizumab	0.55 (0.40–0.77)	**<.001**	0.54 (0.38–0.76)	**<.001**
Salvage tumor-treating fields	0.83 (0.60–1.15)	.267		
*KDR* mutant	1.97 (1.18–3.28)	**.009**	2.51 (1.27–4.94)	**.008**
*KIT* mutant	1.30 (0.81–2.09)	.271		
*PDGFRA* mutant	1.21 (0.83–1.76)	.323		
*RB1* mutant	0.60 (0.38–0.95)	**.028**	0.49 (0.27–0.87)	**.016**
*TP53* mutant	1.28 (0.96–1.70)	.093		

UTHealth, University of Texas Health Science Center at Houston; GBM, glioblastoma; WT, wildtype; KPS, Karnofsky performance status; HR, hazard ratios; CI, confidence interval; GTR, gross total resection; NTR, near-total resection; STR, subtotal resection; TMZ, temozolomide.

*P* ≤ .05 was considered statistically significant and is denoted in bold.

Univariable analysis of OS demonstrated that patients younger than 55 years of age (HR 0.59, *P* < .001), who underwent GTR (HR 0.59, *P* = .028) and NTR (HR 0.52, *P* = .015), who received chemoradiotherapy with TMZ (HR 0.32, *P* < .001), and salvage bevacizumab (HR 0.55, *P <* .001) had a significantly lower risk of death. Meanwhile, patients with a KPS less than 80 (HR 1.63, *P* < .001) had a significantly higher risk of death (Table 1).

**Figure 2.**
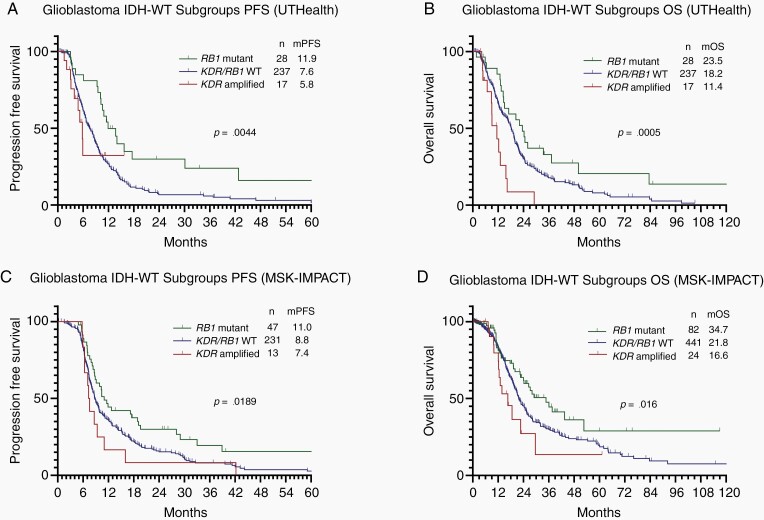
Kaplan–Meier progression-free survival and overall survival of the different molecular subgroups of glioblastoma IDH-WT. (A and B) It demonstrates that *RB1*-mutant patients had the best PFS and OS, *KDR/RB1*-WT patients had an intermediate PFS and OS, and *KDR*-amplified patients had the worse PFS and OS in the UTHealth cohort (*n* = 282). (C and D) It demonstrates that *RB1*-mutant patients had the best PFS and OS, *KDR/RB1*-WT patients had an intermediate PFS and OS, and *KDR*-amplified patients had the worse PFS and OS in the MSK-IMPACT cohort (*n* = 291 – PFS* and *n* = 551 – OS). PFS, progression-free survival; OS, overall survival; GBM, glioblastoma; WT, wildtype; mOS, median overall survival; mPFS, median progression-free survival; UTHealth, University of Texas Health Science Center at Houston; MSK-IMPACT, Memorial Sloan Kettering. Patients who presented concomitant *KDR* amplification and *RB1* alteration were categorized in the *RB1* alteration group. *In the MSK-IMPACT, only 291 patients had PFS available information.

Furthermore, we evaluated the impact of genomic alterations present in at least 3% of the cohort on the OS of GBM IDH-WT patients. The univariable log-rank test showed that GBM IDH-WT patients harboring *RB1* mutations (*n* = 28) had an improved OS compared to *RB1-*WT (*n* = 254) patients (23.5 vs 17.7 months, *P =* .026; Supplementary Figure S2C). Additionally, GBM IDH-WT patients harboring *KDR* amplification (*n* = 20) had worse survival compared to *KDR-*WT (*n* = 262) patients (11.4 vs 18.2 months, *P =* .0008; Supplementary Figure S2D). Notably, *RB1-*mutant and *KDR-*amplified GBM IDH-WT subgroups harbored a distinct PFS and OS, in which *RB1*-mutant patients had doubled the PFS (11.9 vs 5.8 months, *P =* .0691) and OS (23.5 vs 11.4 months, *P =* .0002) compared to *KDR*-amplified GBM IDH-WT (Figure 2A and B).

Multivariable analysis of OS demonstrated that patients younger than 55 years of age (HR 0.63, *P* = 0.010), who received chemoradiotherapy with TMZ (HR 0.24, *P* < .001), received salvage Bevacizumab after progression (HR 0.54, *P* < .001), and harbored an *RB1* mutation (HR 0.49, *P* = .016) had a significantly lower risk of death. Conversely, patients who had a KPS less than 80 (HR 1.44, *P* = .024) and who harbored a *KDR* amplification (HR 2.51, *P* = .008) had a significantly higher risk of death (Table 1). Overall, our institutional cohort shows that GBM IDH-WT can be divided into 3 subgroups with different PFS and OS, by *KDR* and *RB1* status (Figure 2).

### *RB1*-Mutated GBM IDH-WT

Further analysis of GBM IDH-WT tumors by *RB1* status (*RB1* mutant *n* = 28 and *RB1* WT *n* = 254) demonstrated no demographic or clinical differences between the groups. *RB1-*mutant patients were defined as those presenting alterations considered as pathogenic according to COSMIC database as previously described,^16,17^ of which most 27 of 28 (96%) cause loss of function of the gene; meanwhile, the other patient had a p.D697E mutation, which has been previously confirmed as a somatic mutation (COSM7765412). *RB1-*mutant GBMs less frequently harbored *CDKN2A/B* (*RB1* mutant 14% vs *RB1* WT 76%, *P <* .001, *q* < 0.001) and *EGFR* alterations (*RB1* mutant 7% vs *RB1* WT 51%, *P <* .001, *q* < 0.001). Meanwhile, they had increased frequency of *SETD2* (*RB1* mutant 18% vs *RB1* WT 3%, *P =* .003, *q* = 0.006) and *TP53* mutations (*RB1* mutant 75% vs *RB1* WT 26%, *P <* .001, *q* < 0.001; Supplementary Table S1).

### GBM IDH-WT, *KDR* Amplified

Further analysis of GBM IDH-WT tumors by *KDR* status (*KDR* amplified *n* = 20 and *KDR* WT *n* = 262) demonstrated no demographic or clinical differences between the groups. However, *KDR*-amplified tumors were significantly associated with increased *TP53* (*KDR* amplified 60% vs *KDR* WT 28%, *P =* .005, *q* = 0.04) mutations and amplifications in *KIT* (*KDR* amplified 95% vs *KDR* WT 2%, *P <* .001, *q* < 0.001) and *PDGFRA* (*KDR* amplified 100% vs *KDR* WT 8%, *P <* 0.001, *q* < .001; Supplementary Table S1).

### Validation of Findings With the MSK-IMPACT Dataset

**Figure 3.**
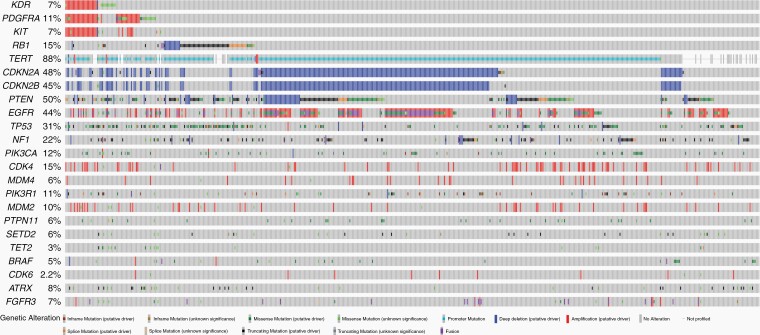
Oncoplot representing the genomic landscape of GBM IDH-WT from the MSK-IMPACT study (*n* = 551).

To validate our findings, we evaluated the MSK-IMPACT GBM IDH-WT dataset (*n* = 551). Eighty (14.5%) patients harbored *RB1* mutations in this dataset. Consistent with our findings, *RB1* mutation was associated with a significantly longer PFS (11.0 vs 8.7 months, *P =* .009) and OS (34.7 vs 21.7 months, *P =* .016; Supplementary Figure S3). Additionally, *RB1* mutations concomitantly occurred with *TP53* (*q* < 0.001) and were mutually exclusive with *CDKN2A/B* (*q* < 0.001) and *EGFR* amplification (*q* = 0.011) in the MSK-IMPACT GBM IDH-WT data (Figure 3). MSK-IMPACT data also demonstrated a mutual exclusivity of *CDK4* amplification in *RB1-*altered patients (*q* = 0.004) and more frequent *NTRK1* mutations (*q* = 0.0002). In the MSK-IMPACT cohort, 432 of 551 (78%) patients had information regarding the MGMT status. There were no differences in MGMT status between *RB1-*mutant (36.76% MGMT methylated and 63.24% MGMT unmethylated) and *RB1-*wildtype (30.49% MGMT methylated and 69.51% MGMT unmethylated) patients (*P =* .3212).

We also evaluated the *KDR* amplification in the MSK-IMPACT GBM IDH-WT dataset (*n* = 551). Twenty-six (5%) patients harbored *KDR* amplification in this dataset. However, *KDR* was not significantly associated with PFS (7.4 vs 9.0 months, *P =* .176) or OS (16.6 vs 22.8 months, *P =* .118; Supplementary Figure S3). Additionally, *KDR* amplification concomitantly occurred with *KIT* (*q* < 0.001) and *PDGFRA* (*q* < 0.001) amplification in the MSK-IMPACT dataset. Although *TP53* mutations appeared to co-occur more frequently in patients with *KDR* amplification (42% vs 28%), it was not significant after multiple comparison adjustments. Additionally, we did not observe differences in MGMT status between *KDR-*amplified and -WT patients (*P =* .2180).

Furthermore, we evaluated the relationship of *KDR* amplification and survival in GBM IDH-WT, utilizing the data of a published study that evaluated *KDR* amplification through fluorescence in situ hybridization (FISH).^20^ In this dataset, 19 of 142 (13%) GBM IDH-WT tumors had *KDR* amplification. Consistent with our study, *KDR-*amplified patients also had worse survival compared to *KDR-*WT patients (3.6 vs 9.2 months, *P =* .009; Supplementary Figure S4). Remarkably, as observed in the UTHealth cohort, the PFS (11.0 vs 7.4 months, *P =* .0237) and OS (34.7 vs 16.6 months, *P =* .0168) of *RB1*-mutant patients differ dramatically from *KDR*-amplified GBM IDH-WT (Figure 2C and D).

## Discussion

In the present study, we sought to identify molecular subgroups of GBM IDH-WT with prognostic significance. We evaluated 282 patients who underwent comprehensive genomic characterization by an NGS assay, interrogating 205 genes and 26 rearrangements. We have identified that GBM IDH-WT patients with *KDR* amplification had worse survival compared to *KDR*-WT patients. Furthermore, *RB1-*mutant patients had improved survival compared to *RB1-*WT patients. Notably, *KDR-*amplified and *RB1-*mutant patients have distinct genetic alterations. These findings suggest that within the group of IDH-WT GBMs there are molecular subgroups with differences in prognosis.

### Outcomes in GBM IDH-WT

Several studies have demonstrated that the survival of patients with GBM is affected by age, KPS, extent of resection, and chemoradiotherapy with TMZ.^1,2,22,23^ The relationship of age in GBM IDH-WT has been confirmed by studies with comprehensive genetic characterization.^6,10^ Our results further confirmed that younger age and chemoradiotherapy with TMZ improved OS in GBM IDH-WT. In line with prior studies, patients with low preoperative functional status (KPS <80) had worse survival.^23^ In addition, we observed that patients treated with salvage bevacizumab had an improved outcome. However, this finding deserves further study, as patients treated with salvage bevacizumab would inevitably have a lead-time bias to improve survival compared to patients who died without a documented recurrence and who were unable to benefit from this therapy. Despite the lack of survival benefit of bevacizumab in randomized clinical trials,^24,25^ these trials were performed prior to the molecular classification of gliomas. Additional studies are needed to evaluate salvage bevacizumab therapy in molecular subgroups of GBM IDH-WT. A recent report has shown that *EGFR-*amplified and classical GBM subgroups are associated with poor response to bevacizumab in recurrent GBM.^26^

### GBM IDH-WT, *RB1* Mutant

**Figure 4.**
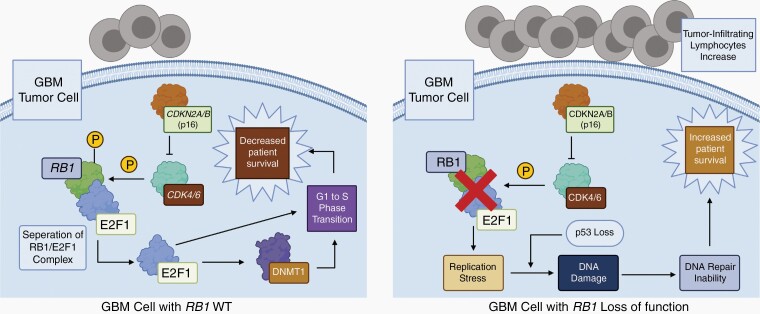
Proposed mechanism of increased survival of GBM IDH-WT, *RB1*-mutant patients based on prior studies. GBM IDH-WT patients with *RB1* loss-of-function mutations have an increased survival through replication stress and DNA damage, which leads to DNA repair inability in the tumor cells. In addition, *RB1* loss of function is correlated with increased tumor-infiltrating lymphocytes (right). These might be the cause of the increased survival compared to *RB1*-WT, GBM IDH-WT patients (left). *RB1* loss of function is represented by the red “X.”

The *RB1* gene was the first tumor-suppressor gene to be molecularly defined.^27^ Dysregulations of the RB pathway signaling are a critical event in gliomagenesis.^28^ In our study, we identified that *RB1* loss-of-function mutations are present in 10% of our institutional cohort and 14.6% of MSK-IMPACT GBM IDH-WT. Interestingly, we observed that *RB1-*mutant GBM IDH-WT patients less frequently harbored *EGFR* alterations and *CDKN2A/B* loss. In addition, *RB1-*mutant cases had a higher frequency of mutations in *TP53* in both the UTHealth and MSK-IMPACT cohorts. Our findings demonstrated that *RB1-*mutant tumors are a subgroup of GBM IDH-WT with a distinct prognosis. *TP53* and *RB1* mutation co-occurrence has been previously reported in several cancers.^7,27^ Also, *RB1* exclusivity with *EGFR* amplification has been demonstrated in GBM xenograft models and glioma patients.^7,28^ Importantly, we identified for the first time that *RB1-*mutant GBM IDH-WT had improved PFS and OS than *RB1-*WT patients after multivariable adjustment (Figure 2 and Table 1). Remarkably, our findings were validated by the MSK-IMPACT cohort. Importantly, these results were not explained by differences in MGMT status in the MSK-IMPACT cohort, as *RB1-*mutant patients’ MGMT status did not differ from *RB1-*WT patients. Loss of the *RB1* gene coupled with a loss in homologous recombination DNA repair pathway genes in other cancers, particularly high-grade ovarian carcinoma, has been associated with increased CD8^+^ tumor-infiltrating lymphocytes (TILs), PFS, and OS.^29^ In GBM, an increase in TILs has been associated with *RB1* mutations.^30^ Additionally, it has been suggested that *RB1* mutations provoke replication stress in tumor cells, leading to DNA damage and activation of the innate immune system. This has been hypothesized to enhance immune checkpoint blockade in ovarian carcinoma.^31^ The DNA damage and increased TILs in *RB1-*mutant patients are plausible explanations for the increased survival in this subtype of GBM IDH-WT (Figure 4). These findings should provoke further studies to identify if *RB1-*mutant GBM IDH-WT might respond more favorably to immune checkpoint inhibitors, a previously failed therapy in this dismal disease.^32^

### GBM IDH-WT, *KDR* Amplified

**Figure 5.**
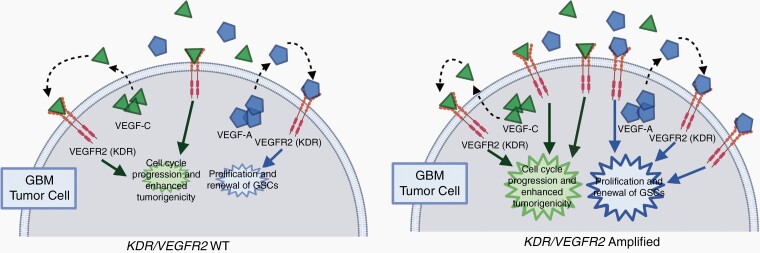
Proposed mechanism for the proliferative effects of *KDR/VEGFR2* amplification in GBM IDH-WT based on prior studies. As seen on the left, binding of VEGF-A and VEGF-C ligands results in increased proliferation of GBM cells. On the right, it is hypothesized that *KDR* amplification would increase the number of available receptors, thus magnifying these effects. In addition, with *KDR* amplification, there may also be an additional elevation of certain protein kinase levels, such as that of c-Met and p38, which serve to increase the invasiveness of the tumor.

*KDR* (*VEGFR2*) is a vascular endothelial growth factor (VEGF) receptor located on the chromosomal 4q11–12 locus, along with *KIT* and *PDGFRA*.^33,34^*KDR* activation by binding of VEGF ligands leads to activation of several downstream oncogenic signaling pathways such as PI3K/AKT, focal adhesion kinase, and mitogen-activated kinase, all resulting in increased cell survival, migration, and angiogenesis.^35^*KDR* alterations are frequently observed in various cancers, including GBM.^33^*KDR* is usually expressed within the tumor endothelium and is the primary VEGF signal transducer, which results in increased cell survival, proliferation, and angiogenesis^20^ (Figure 5). The critical role of VEGF in tumor angiogenesis has been demonstrated in multiple studies. This led to the development of bevacizumab, a monoclonal antibody against the VEGF-A ligand that binds to *KDR*.^36^*KDR* status and its relationship with outcomes have been investigated in GBM.^20,37–39^ However, these studies were performed in a heterogeneous group of patients (IDH-WT and IDH-mutant), prior to the TMZ era, or in relatively small cohorts (Supplementary Table S2). Moreover, recent studies have demonstrated that *KDR* activation through sustained VEGF-C promotes GBM maintenance and growth even under bevacizumab therapy, meaning that activation of *KDR* through VEGF-C is an escape mechanism of GBM to overcome bevacizumab therapy.^40^ The proliferative effects of *KDR* activation in GBM occur with the binding of both VEGF-A and VEGF-C ligands, though it should be noted that the effects of these 2 VEGF ligands have been demonstrated to be non-overlapping (Figure 5). In our study, we identified that *KDR* amplification, which would cause an activation of the *KDR* signaling pathway, correlated to worse outcomes. These findings were further validated with the results of a published GBM IDH-WT study in patients evaluated for *KDR* amplification using FISH.^20^ In contrast to the UTHealth cohort and the published study by Burford et al.,^20^ both demonstrating a statistically significant association with survival, the MSK-IMPACT cohort demonstrated a trend toward shorter survival (16.6 vs 22.8 months) in *KDR*-amplified cases which was not statistically significant.

In addition, autocrine VEGF-C/*KDR* signaling has been shown to regulate cell viability, cell cycle, and in vivo tumor growth in GBM, while autocrine VEGF-A/*KDR* signaling plays a similarly critical role in the proliferation and self-renewal of GBM stem-like cells.^40^*KDR* amplification, accompanied by the presence of more available receptors, would thus serve to exacerbate the effects of such signaling. Along with this, *KDR* amplification has been previously shown in non-small cell lung carcinoma to be associated with VEGF-induced elevated expression of *KDR*, p38, and mTOR pathway components, promoting a more invasive phenotype.^41^ Specifically, it was noted that there was phosphorylation of p38, elevated levels of HIF1α, and increased c-Met activation, correlating with increased angiogenesis and tumorigenesis. Considering these effects together, it would seem to reasonably explain the decreased survival observed in our cohort (Figure 5). Multi-institutional large GBM IDH-WT cohorts are required to further validate the deleterious effect of *KDR* amplification and help identify targeted therapies for this molecular subgroup of GBM IDH-WT. More importantly, survival differences between these molecular subgroups should be considered for clinical trial enrollment to avoid unintended bias.

### Limitations

The limitations of our study include its retrospective design and potential selection bias, as not all GBM IDH-WT patients in our institution underwent NGS. MGMT promoter status for most of the UTHealth cohort was unavailable. However, we did not identify differences in MGMT status by *RB1* or *KDR* mutational status in the MSK-IMPACT cohort. UTHealth and MSK-IMPACT datasets represent different geographic, socioeconomic, and ethnic populations. Moreover, there might be variations in practice patterns between institutions. Additionally, this study did not assess for KDR protein expression levels or TILs. However, we hope that the hypotheses generated by the study results motivate neuro-oncology researchers to identify the mechanisms underlying the survival differences between GBM IDH-WT subgroups. Despite these limitations, our study confirms the association of age, KPS, and chemoradiotherapy (TMZ) with survival in GBM IDH-WT patients. Moreover, we identified molecular subgroups of GBM IDH-WT with prognostic significance in 2 large independent cohorts.

## Conclusions

The current study demonstrates that GBM IDH-WT, *RB1-*mutant represents a different molecular subgroup of GBM with improved PFS and OS. Additionally, we identified that *KDR*-amplified patients had worse survival, which might be related to increased angiogenesis and potential resistance to bevacizumab. Further studies are needed to determine the best treatment strategy for various GBM IDH-WT subgroups, allowing the incorporation of targeted therapies and personalized neuro-oncological care.

## Supplementary Material

vdab050_suppl_Supplementary_Materials

## Notes

**Authorship Statement.** Study design: A.D., L.Y.B., and Y.E.; data recollection: A.D.; data analysis: A.D., A.V.R., and E.W.; manuscript writing: A.D., A.V.R., E.W., M.S., and Y.E.; manuscript revision and editing: A.D., N.T., L.Y.B., and Y.E.; study supervision: L.Y.B. and Y.E.; approved final manuscript: all authors.
