# Supplementary material for: The role of RB1 alteration and 4q12 amplification in IDH-WT glioblastoma
Source: Neurooncol Adv. 2021 Mar 31;3(1):vdab050. doi: 10.1093/noajnl/vdab050 (PMC8193911; doi:10.1093/noajnl/vdab050)
Supplement: vdab050_suppl_Supplementary_Materials [file vdab050_suppl_supplementary_materials.zip › Supplementary Table S2.docx]

**Supplementary Table S2***.* Studies evaluating *KDR* amplification and survival.

| Studies | N of patients | Tumor evaluated | *KDR* amplification | Study mOS | *KDR* amplified mOS | Technique of genomic evaluation | *KDR* amplification relationship with overall survival |
| --- | --- | --- | --- | --- | --- | --- | --- |
| Joensuu et al. 2005 [1] | 47 | GBM | 39 | 9 | 8 | FISH | No association |
| Nobusawa et al. 2011 [2] | 390 | GBM | 3.3 | NA | NA | Differential PCR | No association |
| Burford et al. 2013 [3] | 342 | GBM | 13.7 | 6.5 | 3.6 | FISH | Worse outcome |
| Trevisan et al. 2019 [4] | 113 | GBM | 17.7 | 5.8 | NA | FISH | No association |
| Current Study, 2020 (UTHealth cohort) | 282 | GBM IDH-WT | 7 | 18.0 | 11.4 | NGS | Worse outcome |
| Current Study, 2020 (MSK-IMPACT cohort) | 551 | GBM IDH-WT | 5 | 22.7 | 16.6 | NGS | No association |

Abbreviations. N: number; GBM: glioblastoma; WT: wildtype; mOS: median overall survival in months; FISH: fluorescence in situ hybridization; PCR: polymerase chain reaction NGS: next-generation sequencing; UTHealth: University of Texas Health Science Center at Houston; MSK-IMPACT: Memorial Sloan Kettering Cancer Center.

*Percentage.
