# Supplementary material for: The role of RB1 alteration and 4q12 amplification in IDH-WT glioblastoma
Source: Neurooncol Adv. 2021 Mar 31;3(1):vdab050. doi: 10.1093/noajnl/vdab050 (PMC8193911; doi:10.1093/noajnl/vdab050)
Supplement: vdab050_suppl_Supplementary_Materials [file vdab050_suppl_supplementary_materials.zip › Supplementary Table S1.docx]

**Supplementary Table 1.** Demographic, Clinical, and Genomic Characteristics of Glioblastoma IDH-WT in the UTHealth cohort (n=282).

| Characteristic | | All GBM IDH-WT  N= 282 | *KDR* WT Patients n=262 | KDR amplified patients n=20 | *p-*value | *RB1* WT Patients n=254 | *RB1* Mutated patients n=28 | *p-*value |
| --- | --- | --- | --- | --- | --- | --- | --- | --- |
| Age at diagnosis, median [IQR] | | 61 [53-67.8] | 62.5[61-69] | 60[53-67] | 0.085 | 61 [43-68] | 61.5 [54-66] | 0.855 |
| Male, N (%) | | 170 (60) | 158 (60) | 12 (60) | 1.000 | 158 (62) | 12 (43) | 0.066 |
| Non-Hispanic White, N (%) | | 201 (71) | 185 (71) | 16 (80) | 0.451 | 182 (72) | 19 (68) | 0.664 |
| KPS at Diagnosis 80-100, N (%) | | 107 (38) | 102 (39) | 5 (25) | 0.241 | 95 (37) | 12 (43) | 0.402 |
| 1^st^ Line Therapy, N (%) | |  |  |  |  |  |  |  |
|  | Chemoradiotherapy with TMZ | 258 (91) | 242 (92) | 16 (80) | 0.077 | 231 (91) | 27 (96) | 0.487 |
|  | Bevacizumab | 25 (9) | 22 (8) | 3 (15) | 0.401 | 24 (9) | 1 (4) | 0.487 |
|  | Tumor-Treating Fields | 33 (12) | 32 (12) | 1 (5) | 0.486 | 27 (11) | 6 (21) | 0.115 |
| Surgical Resection, N (%) | |  |  |  |  |  |  |  |
|  | Gross-total resection | 92 (33) | 84 (32) | 8 (40) | 0.879 | 80 (32) | 12 (43) | 0.431 |
|  | Near-total resection | 45 (16) | 42 (16) | 3 (15) |  | 40 (16) | 5 (18) |  |
|  | Subtotal resection | 117 (41) | 110 (42) | 7 (35) |  | 108 (43) | 9 (32) |  |
|  | Biopsy | 27 (10) | 25 (10) | 2 (10) |  | 26 (10) | 1 (4) |  |
| Salvage Therapies* | |  |  |  |  |  |  |  |
|  | Re-Operation | 88 (43) | 86 (45) | 2 (17) | 0.071 | 79 (43) | 9 (45) | 0.810 |
|  | Temozolomide | 94 (46) | 88 (46) | 6 (50) | 1.000 | 85 (46) | 9 (45) | 1.000 |
|  | Bevacizumab | 143 (70) | 136 (71) | 7 (58) | 0.343 | 129 (71) | 14 (70) | 1.000 |
|  | Irinotecan | 78 (38) | 75 (39) | 3 (25) | 0.378 | 69 (38) | 9 (45) | 0.629 |
|  | Lomustine | 17 (8) | 16 (8) | 1 (8) | 1.000 | 16 (9) | 1 (5) | 1.000 |
|  | Tumor-Treating Fields | 63 (31) | 62 (32) | 1 (8) | 0.109 | 56 (31) | 7 (35) | 0.800 |
|  | RT | 50 (25) | 45 (24) | 5 (42) | 0.177 | 46 (25) | 4 (20) | 0.788 |
|  | SRS | 78 (38) | 77 (40) | 2 (17) | 0.133 | 68 (37) | 11 (55) | 0.149 |
| Genetic Alterations, N (%) | |  |  |  |  |  |  |  |
|  | *ATRX* | 8 (3) | 8 (3) | 0 (0) | 1.000 | 8 (3) | 0 (0) | 1.000 |
|  | *BRAF* | 9 (3) | 8 (3) | 1 (5) | 0.489 | 9 (4) | 0 (0) | 0.606 |
|  | *CDK4* | 31 (11) | 27 (10) | 4 (20) | 0.253 | 31 (12) | 0 (0) | 0.054 |
|  | *CDK6* | 8 (3) | 8 (3) | 0 (0) | 1.000 | 8 (3) | 0 (0) | 1.000 |
|  | *CDKN2A/B* | 196 (70) | 184 (70) | 12 (60) | 0.327 | 192 (76) | 4 (14) | **<0.001** |
|  | *EGFR* | 131 (46) | 124 (47) | 7 (35) | 0.355 | 129 (51) | 2 (7) | **<0.001** |
|  | *FGFR3* | 8 (3) | 8 (3) | 0 (0) | 1.000 | 7 (3) | 1 (4) | 0.572 |
|  | *KDR* |  |  |  |  | 17 (7) | 3 (11) | 0.432 |
|  | *KIT* | 24 (9) | 5 (2) | 19 (95) | **<0.001** | 21 (8) | 3 (11) | 0.718 |
|  | *MDM2* | 16 (6) | 15 (6) | 1 (5) | 1.000 | 13 (5) | 3 (11) | 0.204 |
|  | *MDM4* | 26 (9) | 25 (10) | 1 (5) | 1.000 | 24 (9) | 2 (7) | 1.000 |
|  | *NF1* | 44 (16) | 42 (16) | 2 (10) | 0.749 | 38 (15) | 6 (21) | 0.409 |
|  | *PDGFRA* | 40 (14) | 20 (8) | 20 (100) | **<0.001** | 37 (15) | 3 (11) | 0.778 |
|  | *PIK3CA* | 37 (13) | 35 (13) | 2 (10) | 1.000 | 32 (13) | 5 (18) | 0.388 |
|  | *PIK3R1* | 22 (8) | 21 (8) | 1 (5) | 1.000 | 20 (8) | 2 (7) | 1.000 |
|  | *PTEN* | 136 (48) | 126 (48) | 10 (50) | 1.000 | 120 (47) | 16 (57) | 0.328 |
|  | *PTPN11* | 10 (4) | 10 (4) | 0 (0) | 1.000 | 10 (4) | 0 (0) | 0.606 |
|  | *RB1* | 28 (10) | 25 (10) | 3 (15) | 0.432 |  |  |  |
|  | *SETD2* | 12 (5) | 11 (4) | 1 (5) | 0.594 | 7 (3) | 5 (18) | **0.003** |
|  | *TERTp*** | 179 (81) | 168 (64) | 11 (55) | 0.195 | 162 (64) | 17 (61) | 0.772 |
|  | *TET2* | 10 (4) | 10 (4) | 0 (0) | 1.000 | 10 (4) | 0 (0) | 0.606 |
|  | *TP53* | 86 (30) | 74 (28) | 12 (60) | **0.005** | 65 (26) | 21 (75) | **<0.001** |

Abbreviations: UTHealth: University of Texas Health Science Center at Houston TMZ: temozolomide; RT: radiotherapy; SRS: stereotactic radiosurgery. Fischer-exact test and Mann-Whitney U test were used for categorical and continuous variables, respectively. A *p-*value *≤*0.05 was determined as significant (in red). KPS: Karnofsky-performance status. EOR: extent of resection. SRS: stereotactic radiosurgery. GTR: gross-total resection. WT: wild type. *Documented recurrence was available for 203 patients. ***TERTp* mutation was not tested in 61 patients.
