# Supplementary figures and images for: The role of RB1 alteration and 4q12 amplification in IDH-WT glioblastoma
Source: Neurooncol Adv. 2021 Mar 31;3(1):vdab050. doi: 10.1093/noajnl/vdab050 (PMC8193911; doi:10.1093/noajnl/vdab050)

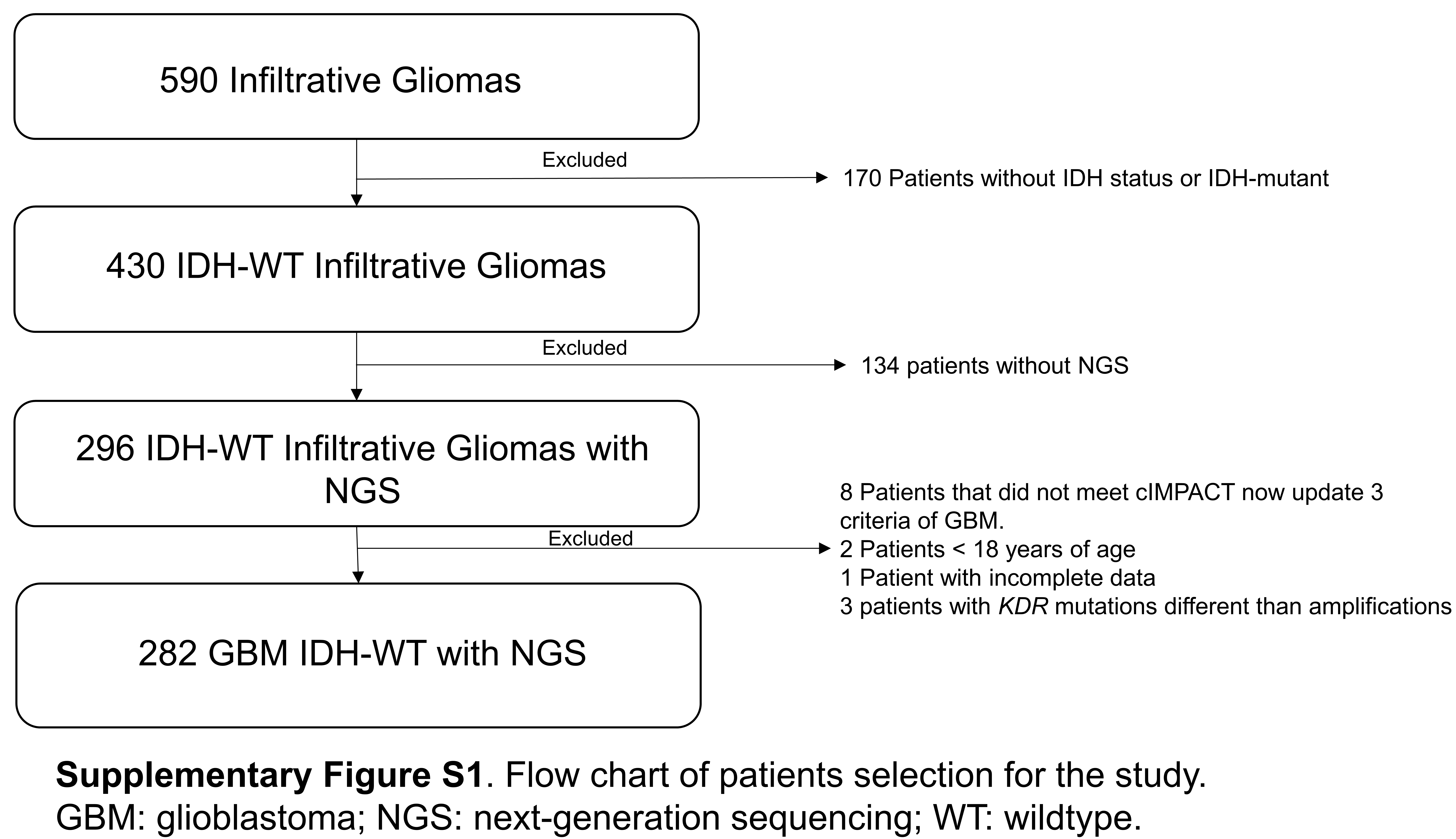

Supplement: vdab050_suppl_Supplementary_Materials [file vdab050_suppl_supplementary_materials.zip › Supplementary Figure S1.tif]

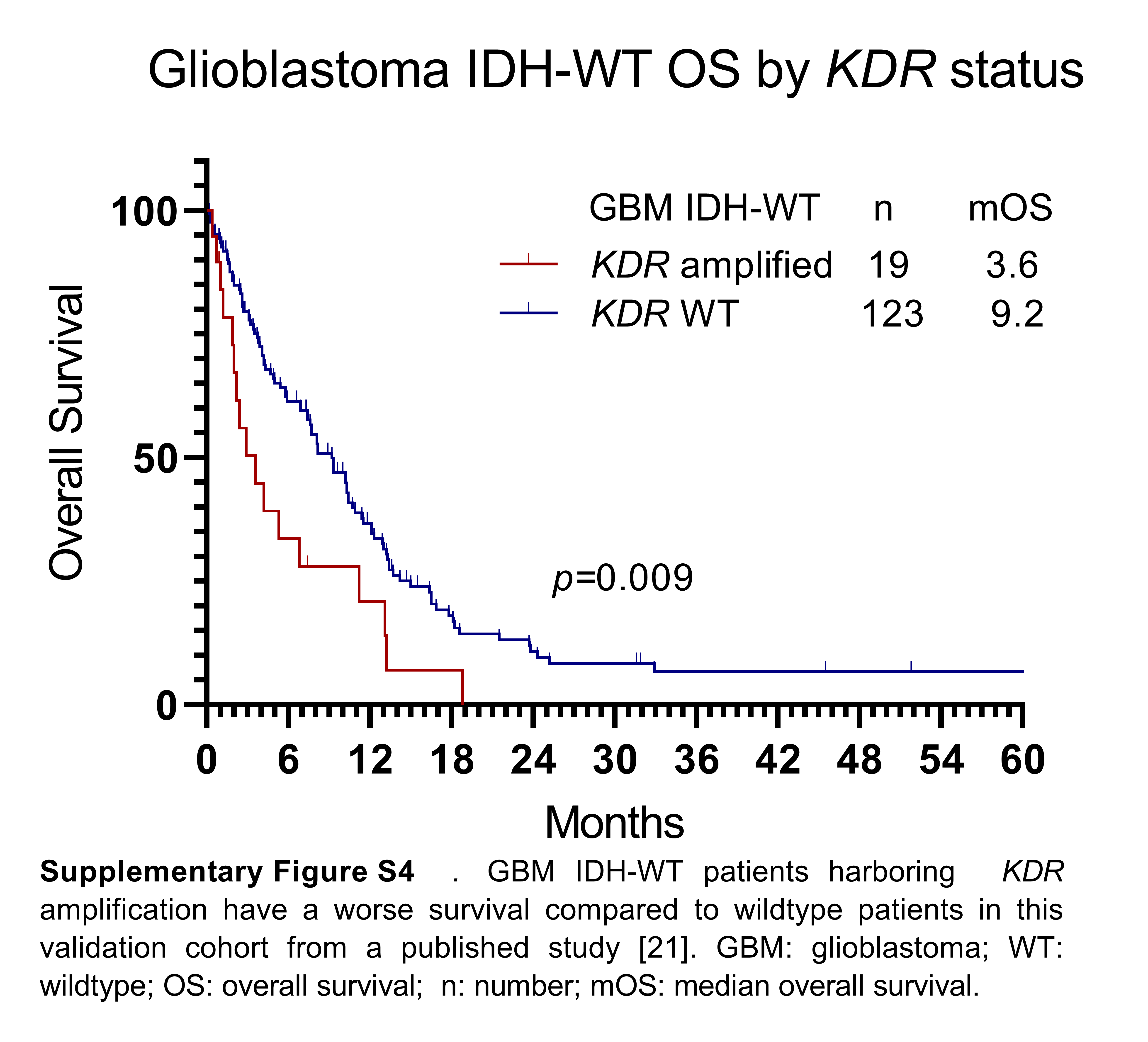

Supplement: vdab050_suppl_Supplementary_Materials [file vdab050_suppl_supplementary_materials.zip › Supplementary Figure S4.tif]
